# Dexamethasone induces docetaxel and cisplatin resistance partially through up-regulating Krüppel-like factor 5 in triple-negative breast cancer

**DOI:** 10.18632/oncotarget.14135

**Published:** 2016-12-24

**Authors:** Zhen Li, Jian Dong, Tianning Zou, Chengzhi Du, Siyuan Li, Ceshi Chen, Rong Liu, Kunhua Wang

**Affiliations:** ^1^ Department of Gastrointestinal and Hernia Surgery, Institute of Gastroenterology, The First Affiliated Hospital of Kunming Medical University, Kunming, Yunnan 650032, China; ^2^ Key Laboratory of Animal Models and Human Disease Mechanisms of Chinese Academy of Sciences & Yunnan Province, Kunming Institute of Zoology, Kunming, Yunnan 650223, China; ^3^ Kunming Digestive Disease Treatment Engineering Technology Center, Kunming, Yunnan 650032, China; ^4^ Department of Oncology, Yunnan Tumor Hospital, The Third Affiliated Hospital of Kunming Medical University, Kunming, Yunnan 650118, China; ^5^ Department of Breast Surgery, Yunnan Tumor Hospital, The Third Affiliated Hospital of Kunming Medical University, Kunming, Yunnan 650118, China

**Keywords:** Dex, GR, KLF5, TNBC, drug resistance

## Abstract

**Purpose:**

Dexamethasone (Dex), a glucocorticoid (GC), is used as a pretreatment drug in cancer patients undergoing chemotherapy. Dex functions by binding to the glucocorticoid receptor (GR) to prevent allergic reactions and severe chemotherapeutic side effects such as nausea and vomiting. However, the mechanisms by which Dex causes chemoresistance remain unknown.

**Methods:**

We used docetaxel and cisplatin to treat triple-negative breast cancer (TNBC) cells with or without Dex and assessed cell proliferation using a sulforhodamine B colorimetric (SRB) assay. Additionally, Western blotting was employed to measure Krüppel-like factor 5 (KLF5), GR and several apoptosis-related proteins. To determine how the GR regulates KLF5, we used qRT-PCR, luciferase reporter assays and ChIP assays. Finally, we detected the involvement of Dex in TNBC chemotherapeutic resistance using HCC1806 xenograft model *in vivo*.

**Results:**

In this study, we demonstrated that Dex induces docetaxel and cisplatin resistance in TNBC cells *in vitro* and *in vivo*. Dex up-regulates pro-survival transcription factor KLF5 expression at both mRNA and protein levels dependent on GR. Importantly, Dex failed to promote cancer cell survival and tumor growth when KLF5 induction was blocked.

**Conclusions:**

We conclude that KLF5 is a Dex-induced gene that contributes to Dex-mediated drug chemoresistance, providing a potential novel target for TNBC treatment.

## INTRODUCTION

Breast cancer is the most common cancer among women in China, accounting for 15% of all newly diagnosed cancers [1]. The incidence of breast cancer was 59.5 per 100,000 people in 2015, and the estimated incidence of breast cancer is expected to reach nearly 100 per 100,000 people in 2021 [2]. According to the molecular classification, triple-negative breast cancer (TNBC) is defined as an estrogen receptor α (ERα)-negative, progesterone receptor (PR)-negative, and human epidermal growth factor receptor-2 (HER2)-negative disease, which represents 12–17% of all breast cancers [3]. Compared with other breast cancer subtypes, TNBC shows a higher rate of recurrence, shorter disease-free survival, and poorer overall survival [4, 5]. Chemotherapy is a standard strategy to treat TNBC patients with metastatic disease. When drug resistance occurs, the lack of effective therapeutics for continuing treatment is a severe problem [6].

Because hormone receptors and HER2 receptors are not expressed in TNBC, no targeted biological agents are available for TNBC treatment [7]. Cytotoxic chemotherapy is the primary systemic treatment recommended under the European Society for Medical Oncology (ESMO) Guidelines [8]. Docetaxel (DTX) is a cytotoxic compound [9] that acts as a semi-synthetic derivative of paclitaxel. Because docetaxel itself is poorly water soluble, it is usually dissolved in Cremophor EL, a polyethoxylated castor oil [10]. However, this solvent frequently causes allergic reactions. Dexamethasone (Dex) is frequently used as a pretreatment drug for patients who take docetaxel, as Dex can prevent allergic reactions and chemotherapeutic side effects such as nausea and vomiting [11, 12]. Dex, a type of glucocorticoid (GC), functions primarily through the ligand-activated nuclear receptor transcription factor glucocorticoid receptor (GR). GCs have been widely used as supportive care medication in the treatment of solid tumors; however, GC treatment may be associated with poor pharmacotherapeutic response or prognosis. Unexpected GC-related mechanisms that cause iatrogenic stimulation of prostate cancer growth have been reported and may contribute to drug resistance and disease progression despite optimal androgen deprivation therapy (ADT) [13]. In addition, GR activation inhibits chemotherapy-induced cell death in high-grade serous ovarian carcinoma [14]. However, the molecular mechanism by which Dex induces chemoresistance has not been fully elucidated.

Krüppel-like factor 5 (KLF5) is a transcription factor that is highly expressed in basal-like breast cancers and may serve as a potential therapeutic target for TNBC [15]. In recent decades, a large number of studies have shown that KLF5 is a key transcription factor that promotes cell proliferation, survival and tumorigenesis [16–18]. The human KLF5 gene encodes a 457 amino acid protein [19]. The Weinberg group reported that high KLF5 expression is one of the nine characteristics of high-level, ER-negative, undifferentiated breast cancers [20]. Breast cancer patients with higher KLF5 expression have poorer prognosis and significantly shorter survival than those with lower KLF5 expression [21, 22]. Previous studies by ourselves and others demonstrated that KLF5 promotes cell proliferation and survival by regulating cyclin D1 [23], FGF–BP [24], mPGES1 [25], and MKP-1 [26], among others. KLF5 is transcriptionally regulated by several hormones, including androgen and progesterone [15, 27], which contribute to hormone-driven tumor progression. We identified several binding sites for hormone receptors, including PR, AR and GR, in the promoter region of the KLF5 gene. Because KLF5 could be regulated by both PR and AR, it may also be regulated by GR.

In this study, we found that KLF5 is induced by the Dex-GR axis, which contributes to Dex-induced drug resistance in TNBC cells. First, we found that Dex promotes cell survival in TNBC treated with chemotherapeutic drugs. Second, we demonstrated that KLF5 is induced by GCs via the GR. Third, we showed that Dex-induced KLF5 expression contributes to Dex-mediated chemoresistance in TNBC cells. Finally, we demonstrated that Dex induces docetaxel resistance in a TNBC xenograft model.

## RESULTS

### Dex induces chemotherapeutic drug resistance in TNBC cell lines

**Figure 1:**
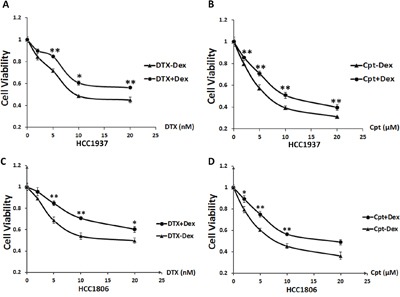
Dex induces chemotherapeutic resistance in the HCC1937and HCC1806triple-negative breast cancer cell lines HCC1937or HCC1806 cells were seeded in 48-well plates at 1.0×10^4^ or 1.5×10^4^ cells/well and treated with the chemotherapeutic drugs docetaxel **A, C**. and cisplatin **B, D**. at the indicated concentrations with or without Dex for 48 h. Cell viability was measured using the SRB assay. *, P<0.05; **, P<0.01.

To determine whether Dex affects cancer cell sensitivity to chemotherapeutic drugs in TNBC cells, we treated the HCC1937 and HCC1806 cell lines with DTX or cisplatin at indicated dosages with or without addition of Dex (10 μM) for 48 hours and measured the cell viability using sulforhodamine B (SRB) assays. As shown in Figure 1A&1B, compared with chemotherapy-only treatment, Dex-treated cells show significantly increased IC_50_ values (DTX: from 9.2 to >20 nM; Cpt: from 6.4 to 10.5 μM) for the drug treatment. Similar results were observed in HCC1806 cells (Figure 1C&1D). Thus, Dex decreased the sensitivity of HCC1937 and HCC1806 cells to DTX and Cpt.

### GCs induce KLF5 expression in TNBC cells and the immortalized breast epithelial cell line MCF10A

**Figure 2:**
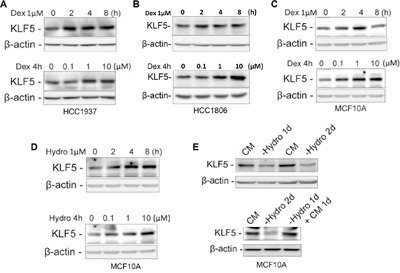
Dexamethasone/hydrocortisone induces KLF5 expression in breast cancer cells **A-C**. KLF5 protein expression is induced by Dex in a time- and dose-dependent manner. HCC1937 (A), HCC1806 (B) or MCF10A (C) cells were treated with 1 μM Dex for the indicated time or for 4 h at the indicated concentrations. **D**. KLF5 protein expression is induced by Hydro in a time- and dose-dependent manner. MCF10A cells were treated with 1 μM Hydro for the indicated times or for 4 hours at the indicated concentrations. **E**. KLF5 expression is regulated by hydrocortisone in MCF10A cells. Removal of Hydro from the MCF10A culture medium down-regulates the KLF5 expression (upper panel), and adding back Hydro restored KLF5 expression (lower panel).

As a crucial factor that promotes TNBC cell proliferation and survival, KLF5 has been reported to play important roles in breast cancer progression. To test the hypothesis that Dex induces drug resistance through KLF5, we first examined whether GCs induce KLF5 expression. HCC1937, HCC1806 and MCF10A cells were treated with two types of GC, hydrocortisone (Hydro) and Dex. We first treated HCC1937 and HCC1806 cells with Dex and found that Dex induced KLF5 expression in a time- and dose-dependent manner (Figure 2A&2B). Similar results were observed in MCF10A cells (Figure 2C). Interestingly, KLF5 was also induced by hydrocortisone in MCF10A cells in a time- and dose-dependent manner (Figure 2D). Because hydrocortisone is a component of the MCF10A culture medium, we wondered whether depletion of hydrocortisone in the MCF10A medium would reduce KLF5 expression. Indeed, as shown in Figure 2E, the KLF5 expression level was dramatically decreased after 1 or 2 days of hydrocortisone depletion from the complete MCF10A culture medium. As expected, adding hydrocortisone back to the culture medium restored KLF5 expression in MCF10A cells. These findings suggest that KLF5 can be induced by GCs.

### Dex induces KLF5 expression through the GR

**Figure 3:**
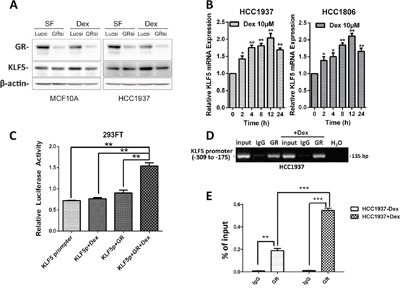
Dexamethasone induces KLF5 transcription via the GR **A**. GR depletion blocks Dex-induced KLF5 expression. Serum-starved (SF) cells were treated with either vehicle control or Dex for 4 h before Western blotting. **B**. Dex induces KLF5 mRNA expression. HCC1937& HCC1806 cells were serum-starved overnight followed by treatment with 10 μM Dex for the indicated time. KLF5 mRNA expression was measured by qRT-PCR. **C**. Dex activates the KLF5 promoter through the GR. 293FT cells were transfected with a GR-expression plasmid and the KLF5 promoter luciferase reporter plasmid. One day after transfection, the cells were treated with 10 μM Dex for 12 h. Cell lysates were collected for the dual-luciferase reporter assay. **D**. GR specifically binds to the KLF5 promoter *in vivo*, as determined by ChIP assays. The input DNA and water were used as the positive and negative controls, respectively. E. The binding capacity of GR to the KLF5 promoter region was quantified by real-time quantitative PCR. All data shown in this figure were confirmed through independent replicates. *, P<0.05; **, P<0.01; ***, P<0.001.

Because GCs function primarily through the GR, we investigated whether Dex induces KLF5 expression through GR. We silenced GR expression in HCC1937 and MCF10A cells using a well-characterized GR siRNA and found that KLF5 expression was not obviously changed compared with the lucsi RNA control in serum-free (SF) medium. However, following Dex stimulation, the induction of KLF5 by Dex was almost completely blocked by GR depletion (Figure 3A). These data clearly indicate that the GR is required for KLF5 induction by Dex.

Because GR is a transcription factor, we wondered whether KLF5 was induced by GR at the mRNA level. As shown in Figure 3B, KLF5 mRNA was increased by Dex after a 2-h treatment and peaked at approximately 12 h, suggesting that KLF5 is a Dex early-response gene and a possible direct target of the GR. KLF5 expression was significantly induced by Dex at physiological concentrations (10 μM). These results suggest that Dex increases KLF5 transcription. To further confirm that Dex induces KLF5 transcription via GR expression, we employed a luciferase reporter assay system to determine whether Dex can activate the KLF5 promoter. As expected, KLF5 promoter activity was significantly activated by Dex in the presence of the GR (Figure 3C and supplementary Figure 1). Because Dex activates the KLF5 gene promoter, we analyzed the KLF5 promoter sequence and identified a 20-bp putative glucocorticoid response element (GRE) (-239 to -219: ACGGTGGGGCGGGGCGGGAG) [28]. To determine whether GR binds to this putative GRE, we performed chromatin immunoprecipitation (ChIP) assays in HCC1937 cells. As shown in Figure 3D, anti-GR antibody, but not the control rabbit IgG, specifically immunoprecipitates the promoter region, but not the coding region, of the KLF5 gene. We further confirmed the binding of the GR to the KLF5 promoter region by real-time quantitative PCR (Figure 3E). Dex significantly increased the binding capacity of the GR to the KLF5 promoter. These results indicate that KLF5 is a direct target of the GR.

### The induction of KLF5 by Dex contributes to Dex-mediated drug resistance

**Figure 4:**
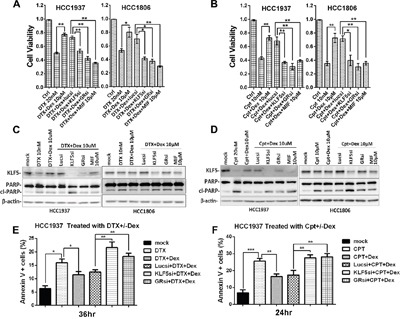
Dex-induced KLF5 contributes to Dex-mediated drug resistance **A &B**. DTX/Cpt-suppressed TNBC cells viability was partially rescued by Dex treatment. Suppression of KLF5 or GR by siRNA or MIF blocked the cell viability increase induced by Dex. **C-F**. DTX/Cpt-suppressed KLF5 expression and induced apoptosis in TNBC cells were partially rescued by Dex treatment. Inhibition of either KLF5 or GR by siRNA or MIF abolished the effect of Dex. *, P<0.05; **, P<0.01; ***, P<0.001.

KLF5 has been shown to promote cell survival, and Dex increases human umbilical vein endothelial cell proliferation and migration [29]. We hypothesized that Dex-induced KLF5 contributes to Dex-mediated drug resistance. To test this hypothesis, we blocked Dex-induced KLF5 expression using a well-characterized KLF5 siRNA and GR siRNA in TNBC cells and examined cell viability. As expected, Dex treatment dramatically increased cell viability, whereas GR depletion blocked not only KLF5 induction but also the Dex-induced cell viability increase. When KLF5 was depleted by KLF5 siRNA, Dex failed to increase cell viability in TNBC cells treated with DTX or Cpt (Figure 4A, 4B and supplementary Figure 2). As shown in Figure 4C and 4D, Dex dramatically decreased the apoptosis (indicated by cleaved PARP) induced by DTX and Cpt treatment in TNBC cells. GR depletion blocked not only KLF5 induction but also the Dex-mediated decrease in apoptosis. Similar to GR depletion, the GR antagonist mifepristone (MIF) also suppressed the Dex-induced KLF5 activation and decrease in apoptosis. We’ve also obtained similar results when we use flow cytometry analysis to test the percentage of apoptosis cells (Figure 4E&4F).

In order to test whether the induction of KLF5 expression by Dex also contributes to Dex-induced mediated drug resistance in other types of breast cancer cells other than TNBC, we treated ER-positive breast cancer cell MCF7 with Dex and found KLF5 expression indeed is induced by Dex in a time course-dependent manner (supplementary Figure 2C). Meanwhile, we also found Dex treatment does cause chemotherapeutic resistance in MCF7 cells (supplementary Figure 2D).

**Figure 5:**
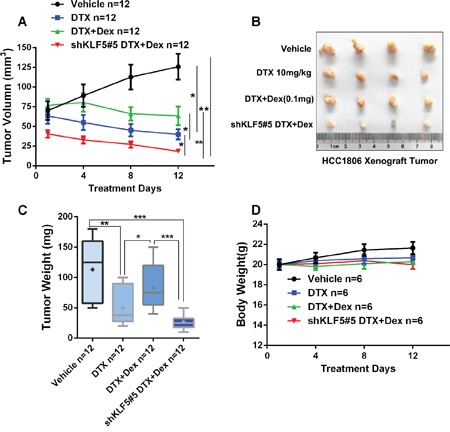
KLF5 contributes to Dex-mediated drug resistance *in vivo* **A-C**. KLF5 depletion blocked Dex-induced DTX resistance *in vivo*. 1 × 10^6^ HCC1806 control or KLF5 depleted cellswere injected to female nude mice subcutaneously. When the tumor size reached around 70 mm^3^, mice were treated with injected with 10 mg/kg DTX with or without 1mg/ml Dex. **D**. DTX or Dex treatment does not significantly affect body weight of nude mice.

In order to further evaluate whether Dex induces chemotherapeutic resistance via regulating KLF5 *in vivo*, we stably depleted KLF5 in HCC1806 and injected theses cells or control cells to immunodeficient nude mice subcutaneously. As shown in Figure 5, DTX significantly suppressed tumor growth compared to the control group; however, together with Dex, the anti-tumor effects of DTX was significantly impaired. Interestingly, depletion of KLF5 blocked Dex-caused chemotherapeutic resistance *in vivo*. Taken together, these findings suggest that KLF5 contributes, at least partially, to Dex-mediated chemotherapeutic drug resistance.

## DISCUSSION

Breast cancer is an important health concern for women, especially TNBC, which is among most malignant subtypes of breast cancers. TNBC lacks the expression of either ER or Her2, meaning that endocrine therapy that targets the ER and targeted therapy against Her2 are ineffective in TNBC. Therefore, the predominant clinical strategy for TNBC patients is systemic chemotherapy. However, the development of chemoresistance is another important problem in the treatment of breast cancers. In this study, we focused primarily on investigating whether the clinical use of Dex in combination with chemotherapeutic drugs (such as DTX and Cpt) causes chemoresistance *in vitro and in vivo*, as well as the possible mechanisms.

Dex reduces the sensitivity of TNBC cells to chemotherapeutic drugs. DTX, a semi-synthetic analogue of paclitaxel, belongs to the taxane class of drugs. DTX predominantly functions by altering microtubule assembly, which inhibits mitotic cell division and causes cancer cell death. DTX is currently one of the best single-use drugs for the treatment of advanced breast cancer, as it not only resides in the cells for a long period of time but also has an intracellular concentration that is three times higher than that of paclitaxel [30]. As a result, DTX is used as either a first- or second-line drug in the clinical treatment of breast cancer and shows no obvious cross-resistance with paclitaxel [31, 32]. Reports suggest approximately that 23 - 65% of breast cancer patients who fail to respond to first-line drug treatment still show sensitivity to DTX-only treatment [33, 34]. Cisplatin, a platinum metal complex, causes cytotoxic effects predominantly through the formation of platinum-DNA adducts independent of cell cycle phase. Cpt primarily induces DNA damage and inhibits cell division [35]. However, these widely used chemotherapeutic drugs also cause severe side effects.

GCs such as Dex are conventionally used to help cancer patients reduce nausea, improve appetite, relieve bone pain and inhibit edema while undergoing radiation and chemotherapy [36]. However, recent studies found that GCs may induce resistance to chemotherapeutic drugs in a variety of solid tumors [37]. GCs have been shown to inhibit apoptosis in liver, esophageal, colorectal, bladder, kidney and prostate cancer cells [38]. Dex has also been shown to decrease apoptosis induced by chemotherapeutic drugs [39]. GCs are also responsible for resistance to chemotherapy and radiotherapy in multiple malignant solid tumors [40]. In the present study, we demonstrated that Dex also reduced the cytotoxicity of DTX and Cpt in TNBC cells (Figure 1). However, the mechanism responsible is unknown.

We demonstrated that the Dex-GR-KLF5 axis contributes to the chemoresistance of TNBC cells. Dex is widely used in combination with chemotherapeutic drugs to reduce side effects. Chemoresistance caused by Dex is an urgent problem that needs to be resolved. Dex can induce the expression of MAPK phosphatase 1 (MKP1), which inhibits apoptosis induced by chemotherapeutic drugs such as paclitaxel [41]. We previously found that KLF5 promotes breast cancer cell survival by stabilizing the MKP1 protein [26]. Additionally, KLF5 has also been reported to increase drug resistance in ovarian cancer cells by inducing survivin gene expression [42]. We tested whether KLF5 mediated the chemoresistance effects of Dex in TNBC cells. Indeed, as shown in Figure 4A&4C and Figure 5, KLF5 depletion restored TNBC cells sensitivity to DTX and Cpt.

GCs are steroid hormones that are secreted by the adrenal cortex and include cortisol and corticosterone. GRs are important members of the nuclear receptor super family. GRs are typical hormone-dependent transcription factors that regulate target gene transcription by binding to GREs in target genes. We found that KLF5 was induced by GCs in HCC1937 cells in a GR-dependent manner (Figure 2 and 3). The ChIP assay showed that GR specifically immunoprecipitates the promoter region of the *KLF5* gene. As shown in Figure 4A and 4C, the chemotherapeutic drug-induced loss of cell viability was largely restored by Dex treatment. Because Dex promotes TNBC cell survival following chemotherapeutic drug treatment, it is possible that blocking Dex-GR signaling can reverse these effects. Indeed, GR blockade by either siRNA or the GR antagonist MIF re-sensitized TNBC cells to chemotherapeutic drugs. MIF, a clinically used contraceptive drug, has a strong affinity for the PR and GR. Previously, we demonstrated that MIF can block KLF5 induction by progesterone in PR-positive breast cancer cells [15] and down-regulate KLF5 expression in TNBC cells by inducing miR-153 expression [43]. By competitively binding to these receptors, MIF induces progesterone resistance and GC resistance [44, 45]. Our studies suggest that MIF may have clinical applications in reducing Dex-induced chemoresistance.

In conclusion, we determined that KLF5 is induced by Dex via GR in TNBC cells. Dex-induced KLF5 contributes to Dex-mediated chemoresistance *in vitro and in vivo*. Importantly, we found that blockade of the GR-KLF5 axis re-sensitizes TNBC cells to chemotherapeutic drugs. Together these findings suggest that Dex may cause chemoresistance by inducing KLF5 in TNBC.

## MATERIALS AND METHODS

### Cell culture and transfection

Human TNBC cells (HCC1937 and HCC1806), immortalized breast epithelial cells (MCF10A) and transfected- human renal derived 293 cells (293FT) were purchased from ATCC. HCC1937 and HCC1806 were cultured in RPMI-1640 supplemented with 10% FBS (HyClone, Utah, USA). MCF10A was cultured in DMEM-F12 (HyClone, Utah, USA) media supplemented with 2 mM L-glutamine, 1% penicillin/streptomycin, 200 ng/ml EGF, 100 ng/ml cholera toxin, 0.01 mg/ml insulin, 500 ng/ml hydrocortisone, and 5% horse serum (Gibco, California, USA). 293FT was cultured in DMEM supplemented with 10% FBS (HyClone, Utah, USA).

All plasmids and siRNAs were transfected using Lipofectamine 2000 (Invitrogen, Carlsbad, CA). The KLF5 siRNA has been described in our previous study [24]. The target sequence of the GR siRNA is 5 ’ -GCACCTTTGACATCTTGCAGGATTT-3’.

### Reagents

Chemicals (Dex, DTX, and mifepristone) and the anti-β-actin antibody were purchased from Sigma (St. Louis, MO). Cpt was purchased from Qilu Pharmaceutical, Co. (San Dong, China). The anti-KLF5 antibody has been described previously [46]. The anti-GR antibody was purchased from Santa Cruz Biotechnology, Inc. (Santa Cruz, CA). The anti-PARP, anti-caspase 3 and anti-caspase 7 antibodies were purchased from Cell Signaling Technology (Danvers, MA).

### Sulforhodamine B colorimetric (SRB) assays

SRB assays were performed to measure cell viability. After treatment, the cells were fixed with cold 10% trichloroacetic acid (TCA) and stained with SRB (Sigma). Excess dye was washed out using 1% acetic acid. The protein-bound dye was dissolved in 10 mM unbuffered Tris-base solution, and the OD_530_ was read on a microplate reader (Epoch, BioTek).

### Western blotting

Cells were washed with cold 1×PBS and collected with lysis buffer (50 mM Tris-Cl, pH 7.4, 150 mM NaCl, 1 mM EDTA, 1% Triton X-100). Equal amounts of protein lysates were loaded for Western blot analysis. Images were acquired using an Image Quant® LAS 4000 (GE HealthCare, Pittsburgh, PA).

### Quantitative RT-PCR

HCC1937 and HCC1806 cells were treated with 10μM Dex for the indicated times. Total mRNA was isolated using TRIzol® reagent (Invitrogen). Reverse transcription was performed using the iScript cDNA Synthesis Kit (Bio-Rad, CA), and cDNAs were generated using the Real-Time SYBR Green PCR master mix on an ABI-7900 system (Thermo Fisher Scientific, Inc., Pittsburgh, PA). The follows primers were used: GAPDH, forward: 5’ -GGTGAAGGTCGGAGTCAACG-3’ and reverse: 5’ -TGGGTGGAATCATATTGGAACA-3’; KLF5, forward: 5’ -ACACCAGACCGCAGCTCCA-3’ and reverse: 5’ -TCCATTGCTGCTGTCTGATTTGTAG-3’.

### Dual luciferase assays

293FT cells were plated in 24-well plates at a density of 1.5×10^5^ cells per well in triplicate. After 24 h, the cells were transfected with 800 ng KLF5 promoter reporter plasmid and an internal control pRL-TK plasmid together with the GR expression plasmid or vector control using Lipofectamine 2000 (Invitrogen). The 1.9-kb human KLF5 gene promoter and the GR plasmid have been described previously [28]. One day after transfection, the cells were serum starved overnight, followed by treatment with 10 μM Dex for another 12 h. Luciferase activities were measured using the dual luciferase reporter assay kit (Promega, Madison, WI) following the manufacturer's instructions on an Infinite200 (TECAN, Switzerland).

### Chromatin immunoprecipitation assay

Serum-starved HCC1937 cells were treated with 10 mM Dex or vehicle for 12 h before collection. The ChIP assay was performed following a protocol provided by Abcam (Cambridge, MA, USA). The diluted DNA-protein complex was incubated overnight with an equal amount of either anti-GR antibody or IgG control (Santa Cruz, CA, USA) at 4°C in the presence of protein A/G beads. After immunoprecipitation, chromosomal DNA was purified and analyzed using PCR to detect the predicted KLF5 promoter region. The primers used for amplifying the KLF5 promoter region surrounding the potential GRE site (-309 to -175) were 5’ -TACGTGCGCTCGCGGTTCTCT-3’ and 5’ -TCCGCTCTTCCACACGTA-3’.

### Annexin V assay

HCC1937 cells were seeded in 6-well plates at a density of 3.5×10^5^ cells per well in triplicates. One day after, the cells were transfected with siRNAs and treated with DTX (10nM) or Cpt (10μM) with or without Dex for 36h or 24h, respectively. Cells were collected and stained with anti-Annexin V antibody (eBioscience) and analyzed on an Accuri C6 flow cytometer (BD bioscience, San Diego, USA).

### Tumorigenesis in immunodeficient nude mice

Six-week-old female nude mice were purchased from Hunan SJA Laboratory Animal Co., Ltd. The mice were maintained in a barrier unit with a 12 h light−dark switch. Freshly harvested HCC1806 cells (control or KLF5 depleted cells) (1 × 10^6^ cells per point, resuspended in 100 μl PBS) were injected subcutaneously. When the tumor size reached around 70 mm^3^, the control HCC1806 injected mice were randomly and equally distributedinto three groups (n = 6/each group), which were injected intraperitoneally with 10 mg/kg DTX with or without 1mg/ml Dex. Tumor size and mouse weight were measured every three days. The animal protocol was approved by the Animal Care and Use Committee at Kunming Institute of Zoology, Chinese Academy of Sciences.

### Statistical analysis

All experiments were repeated at least three times. The values are expressed as the mean ± standard deviation (S.D.) and were analyzed using Student's t-test. Error bars show SD values from triplicates in a single representative experiment. P values less than 0.05 were considered significant.

## SUPPLEMENTARY MATERIALS FIGURES AND TABLES


