# Supplementary material for: Dexamethasone induces docetaxel and cisplatin resistance partially through up-regulating Krüppel-like factor 5 in triple-negative breast cancer
Source: Oncotarget. 2016 Dec 24;8(7):11555–65. doi: 10.18632/oncotarget.14135 (PMC5355285; doi:10.18632/oncotarget.14135)
Supplement: Supplementary file 1 [file oncotarget-08-11555-s001.pdf]

Dexamethasone induces docetaxel and cisplatin resistance partially through up-regulating Krüppel-like factor 5 in triple-negative breast cancer

SUPPLEMENTARY FIGURES

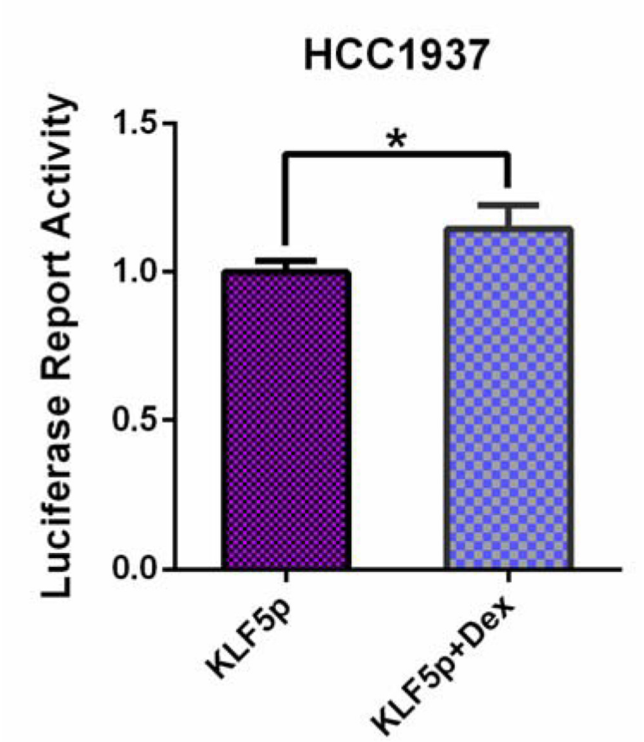

Supplementary Figure 1: Dex activates the KLF5 promoter through the GR. HCC1937 cells were transfected with KLF5 promoter luciferase reporter plasmid together with β-actin-Renilla control plasmid. One day after transfection, the cells were treated with or without 10 μM Dex for 12 h. Cell lysates were collected for the dual-luciferase reporter assay. *, $P<0.05$.

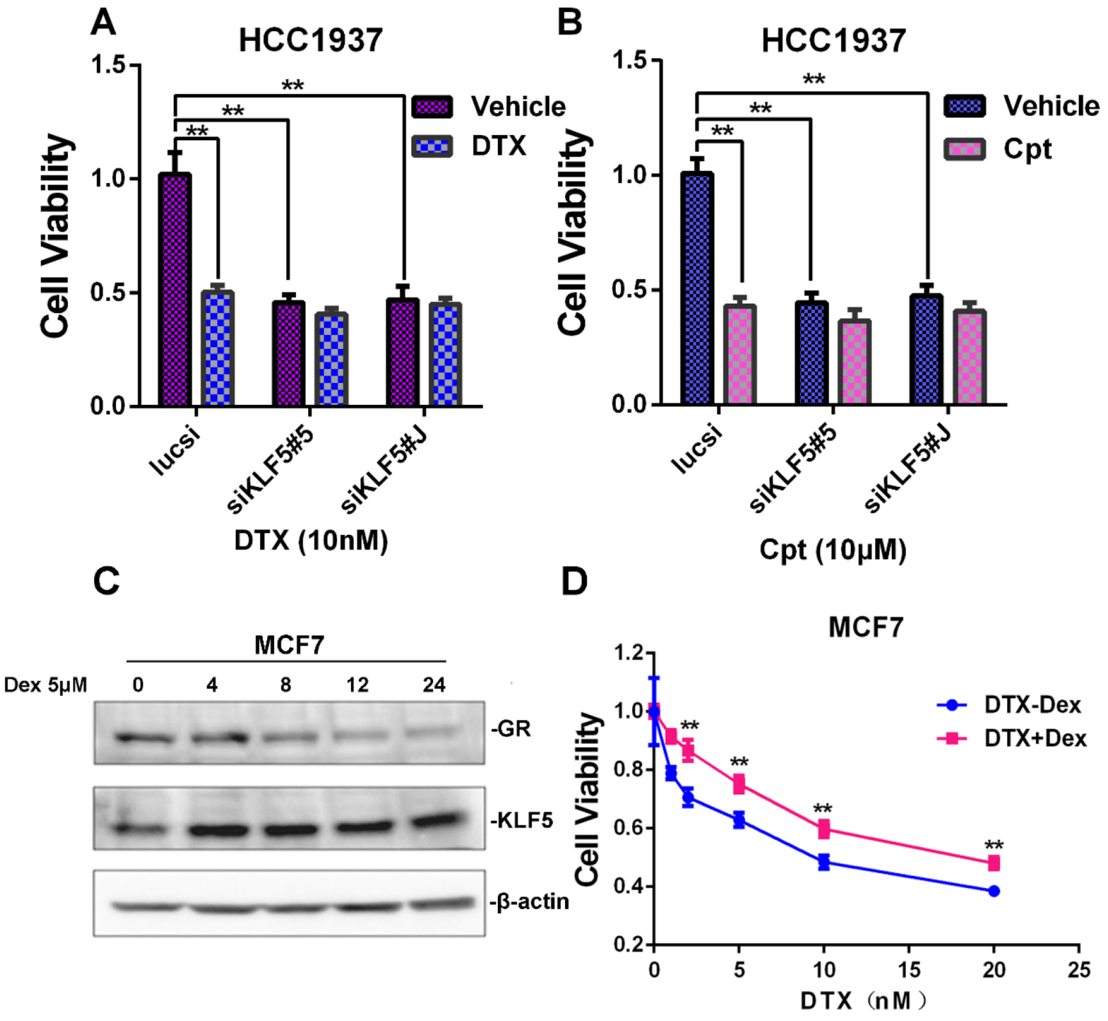

Supplementary Figure 2: The induction of KLF5 plays important roles in TNBC cell survival. **A & B.** HCC1937 was transfected with KLF5#5 or #J siRNA and cell viability was examined by SRB Assay. Depletion of KLF5 by two different siRNAs significantly decrease the cell viability compared to control siRNA group, and these effect are similar as suppression of DTX. **C.** KLF5 is induced in MCF7 breast cancer cells. MCF7 cell was with 5 μM Dex for indicated time. **D.** Dex induces chemotherapeutic resistance in MCF7 cell line. MCF7 cells were seeded in 48-well plates at 3.0×10^4 cells/well and treated with docetaxel at indicated dosages with or without Dex for 48 h. Cell viability was measured using the SRB assay. **, P<0.01.
